# Estratificação da Insuficiência Cardíaca Clínica através do Mapa T1 Nativo: Experiência de um Serviço de Referência

**DOI:** 10.36660/abc.20190782

**Published:** 2021-05-06

**Authors:** Thiago dos Santos Silva Marques, André Maurício de Souza Fernandes, Roberto Nery Dantas, Robert W. Biederman, Ana Paula Marques de Oliveira Melo, Roque Aras

**Affiliations:** 1 Universidade de São Paulo Faculdade de Medicina Hospital das Clínicas São PauloSP Brasil Instituto do Coração do Hospital das Clínicas da Faculdade de Medicina da Universidade de São Paulo, São Paulo, SP - Brasil; 2 Universidade Federal da Bahia Faculdade de Medicina de Bahia SalvadorBA Brasil Universidade Federal da Bahia - Faculdade de Medicina de Bahia, Salvador, BA - Brasil; 3 Allegheny General Hospital PittsburghPennsylvania EUA Allegheny General Hospital, Pittsburgh, Pennsylvania - EUA; 4 Hospital Heliópolis São PauloSP Brasil Hospital Heliópolis, São Paulo, SP - Brasil; 5 Hospital Universitário Professor Edgard Santos SalvadorBA Brasil Hospital Universitário Professor Edgard Santos, Salvador, BA - Brasil

**Keywords:** Insuficiência Cardíaca, Cardiomiopatia Dilatada, Disfunção Ventricular Esquerda, Fibrose, Diagnóstico por Imagem, Cardiomiopatia Chagásica, Espectroscopia de Ressonância Magnética/métodos

## Abstract

**Fundamento:**

Fibrose cardíaca difusa é fator importante na avaliação prognóstica dos pacientes com disfunção ventricular. Mapeamento T1 nativo pela ressonância magnética cardíaca (RMC) apresenta elevada sensibilidade e é considerado preditor independente de mortalidade por todas as causas e desenvolvimento de insuficiência cardíaca (IC) nos pacientes com cardiomiopatia.

**Objetivos:**

Avaliar aplicabilidade da avaliação com mapa T1 nativo em pacientes com IC em um hospital de referência de cardiologia e sua associação com parâmetros estruturais e perfil funcional.

**Métodos:**

Estudo transversal com pacientes adultos com IC classes funcionais NYHA I e II, isquêmicos e não isquêmicos, acompanhados em hospital de referência, que realizaram RMC. Os valores de T1 nativo foram analisados em relação a parâmetros estruturais, comorbidades, etiologia e categorização da IC pela fração de ejeção do ventrículo esquerdo (FEVE). Análises foram realizadas com nível de significância de 5%.

**Resultados:**

Analisados 134 pacientes. Valores de T1 nativo elevados foram encontrados em pacientes com maior dilatação (1004,9 vs 1042,7ms, p=0,001), volume (1021,3 vs 1050,3ms, p<0,01) e disfunção ventricular (1010,1 vs 1053,4ms, p<0,001), mesmo quando analisados isoladamente os não isquêmicos. Pacientes classificados com IC com fração de ejeção reduzida apresentaram maiores valores T1 em relação aos com IC e fração de ejeção preservada (ICFEP) (992,7 vs 1054,1ms, p<0,001). Dos com ICFEP, 55,2% apresentavam T1 elevado.

**Conclusões:**

Mapeamento T1 por RMC é factível para avaliação da IC clínica. Houve associação direta entre maior valor nativo de T1 e menor fração de ejeção, maiores diâmetros e volumes do VE, independentemente da etiologia da IC.

## Introdução

Fibrose cardíaca tornou-se fator importante na avaliação prognóstica dos pacientes com disfunção ventricular, sendo considerada como uma das consequências do remodelamento patológico do ventrículo esquerdo (VE),^1^ que desempenha um marcante papel na resposta do miocárdio à lesão. Quando em excesso leva à progressão da insuficiência cardíaca (IC) e pior prognóstico.^2^ Métodos de imagem não invasivos para avaliação quantitativa, em um estágio inicial, da presença e extensão da fibrose miocárdica são necessários para melhor estratificação de risco de IC e para monitorar os efeitos do tratamento.^3^

Ressonância magnética cardíaca (RMC), considerada ferramenta eficaz para avaliar a morfologia e função miocárdica, bem como alterações teciduais,^4^^–^^7^ surgiu como uma modalidade não invasiva de primeira linha para investigação de etiologia e prognóstico em pacientes com disfunção miocárdica.^8^^,^^9^ O mapeamento T1 nativo é um método rápido e sem contraste que visa detectar alterações miocárdicas difusas, em uma variedade de condições cardíacas. Apresenta ampla sensibilidade comprovada para alterações patológicas, incluindo detecção de edema miocárdico, infarto, isquemia, cardiomiopatias e fibrose difusa.^10^^–^^14^ Portanto, o mapeamento T1 nativo é um método de imagem alternativo para avaliação da área cardíaca de risco.^15^

Estudo observacional multicêntrico evidenciou que o T1 nativo mostrou ser melhor preditor expressivo de piores desfechos na cardiomiopatia dilatada (CMD) do que os parâmetros clínicos clássicos, mostrando que T1 nativo foi o mais forte preditor independente de mortalidade por todas as causas e desenvolvimento de IC.^16^^,^^17^ A gravidade de doença difusa, avaliada pelo mapa T1 nativo, pode ser fisiopatologicamente um parâmetro mais relevante, pois está diretamente relacionada à progressão da doença e à capacidade funcional do miocárdio remanescente. A natureza contínua dos valores de T1 corresponde bem à taxa de eventos clínicos: quanto maior o T1 nativo, maior o risco de eventos adversos e vice-versa. Esses achados permitem refinar a abordagem atual de estratificação de risco em pacientes com cardiomiopatias, principalmente na CMD.^17^

Nosso estudo objetiva avaliar a aplicabilidade da avaliação com mapa T1 nativo em pacientes com IC em um hospital de referência de cardiologia e sua associação com parâmetros estruturais e perfil funcional destes pacientes.

## Métodos

### Estudo e Amostra

Foram incluídos pacientes acompanhados no ambulatório de IC do Hospital Ana Nery, Salvador, Bahia, que consecutivamente foram encaminhados para realização de RMC como parte do cuidado clínico e diagnóstico no período compreendido entre os anos de 2012 e 2016.

Foram selecionados, de forma consecutiva, pacientes com idade ≥ 18 anos com diagnóstico de IC, de acordo com critérios de Framingham e/ou Boston, conforme Diretriz Brasileira de Insuficiência Cardíaca Crônica e Aguda, com classes funcionais I e II pela *New York Heart Association* (NYHA), com ao menos IC diastólica tipo II definida pelo ecocardiograma transtorácico, de diversas etiologias, divididas em isquêmicas ou não isquêmicas, definição essa baseada na documentação de infarto do miocárdio (IM) prévio, pela presença de realce tardio pelo gadolínio (RTG) subendocárdico ou transmural (respeitando território coronariano) na RMC e/ou presença de isquemia por algum método diagnóstico. Em relação à cardiomiopatia chagásica, foi considerado o diagnóstico na presença de sorologia positiva e após exclusão de isquemia.

Todos os pacientes realizavam exames de rotina no ambulatório de IC, como radiografia de tórax, teste de caminhada e eletrocardiograma, associado a avaliação de uma equipe multiprofissional. Todos pacientes eram acompanhados no serviço de IC da unidade e faziam uso de terapia medicamentosa otimizada, associada ou não à reabilitação cardíaca pela equipe multidisciplinar, conforme critério clínico do médico assistente.

O trabalho foi aprovado pelo Comitê de Ética e Pesquisa da instituição, como subprojeto do trabalho principal: “Características dos pacientes submetidos à ressonância magnética cardiovascular em um hospital de referência”.

### Protocolo de Aquisição e Avaliação de Exames de RMC

Todos os exames de RMC foram realizados em um scanner de corpo inteiro Avanto de 1,5 T (Siemens Medical Solutions, Alemanha) usando uma bobina cardíaca de 8 canais. Imagens em varredura foram realizadas para obter sequências cine SSFP em duas, três e quatro câmaras, além do eixo curto. As imagens de cine foram adquiridas durante apneia expiratória (20 quadros por ciclo cardíaco com cortes de 8mm de espessura, FOV 300, matriz 208 Åx 80, BW 925 KHz/pixel). Para análise da função do VE, o eixo curto foi composto de um mínimo de 8 e um máximo de 12 cortes, com 8 mm de espessura e 2 mm de intervalo.

Imagens de mapa T1 nativo foram realizadas sem injeção de contraste através de corte em porção média do VE através da sequência *Modified Look-Locker Inversion recovery* (MOLLI), com acoplamento eletrocardiográfico, 250 a 360 mm de FOV; 192 × 122 a 192 × 183 de tamanho da matriz. Espessura do corte de 6-8 mm; 2,2 / 1,1ms ≈ TR / TE, ângulo de viragem de 35 °; Fator GRAPPA = 2; 17 batimentos cardíacos (que coletam 3 + 3 + 5 amostras). Como a utilização de contraste nos estudos não fazia parte do protocolo e esteve reservado a critério clínico, não foi realizado o cálculo de volume extracelular (VEC) e mapa T1 pós contraste.

O valor de normalidade do T1 nativo, para nossa amostra, foi obtido previamente através de um estudo piloto com pacientes sem comorbidades e coração estruturalmente normal da mesma instituição/scanner, como recomendado pela Society for Cardiovascular Magnetic Resonance (SCMR).^18^ Segundo resultado dessa avaliação, o valor médio de normalidade considerado para o T1 miocárdico nativo foi de 983,46 ± 34,38 ms.

**Figura 1:**
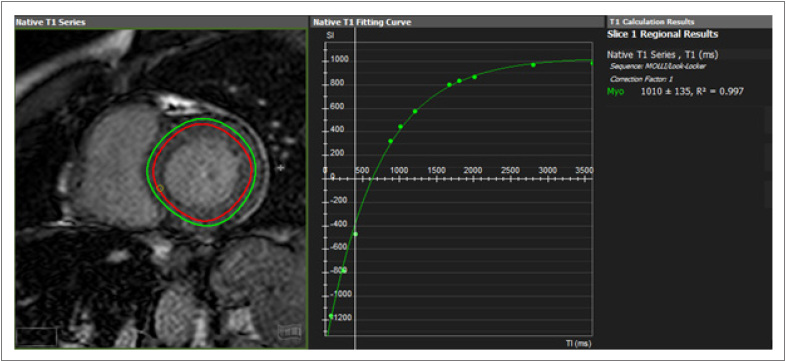
Cálculo do mapa T1 nativo.

Todos os exames foram analisados através do software cvi42 (*Circle Cardiovascular Imaging Inc*., Calgary, Canada) por especialista em imagem cardiovascular com mais de 5 anos de experiência. Após delineamento de bordos endocárdicos e epicárdicos no eixo curto do VE, em sístole e diástole máximas, foram quantificadas variáveis funcionais como fração de ejeção do ventrículo esquerdo (FEVE), diâmetro diastólico final do ventrículo esquerdo (DDFVE), volume diastólico final do ventrículo esquerdo (VDFVE), massa cardíaca (indexados para superfície corpórea), avaliados conforme valores de referência em RMC recomendados.^19^ Para cálculo do mapa T1 nativo, os bordos dos traçados foram realizados de forma estreita com intuito de evitar ao máximo contaminação com a cavidade ventricular ou com gordura epicárdica, e de forma a evitar áreas com realce tardio miocárdico identificável visivelmente (Figura 1). Os exames foram analisados por um único profissional experiente.

Fonte: Marques, 2019.

Os valores de T1 miocárdico nativo obtidos foram analisados em relação às comorbidades clínicas, parâmetros estruturais, etiologia e categorização da IC. A IC foi categorizada em: 1) FEVE reduzida (IC de fração de ejeção reduzida – ICFER: < 40%); 2) FEVE limítrofe (IC de fração de ejeção limítrofe – ICFEL: 40 a 49%); e 3) FEVE preservada (IC de fração de ejeção preservada – ICFEP: ≥ 50%) (20,21).

### Análise Estatística

Os dados coletados foram descritos através de médias e desvio padrão para as variáveis contínuas de distribuição normal; e medianas e intervalo interquartílico para as de distribuição não normal. As variáveis categóricas foram descritas em números absolutos e porcentagens. A normalidade das variáveis foi testada através do teste de Kolmogorov-Smirnov. Foram realizados testes estatísticos, conforme o tipo de variável e a normalidade da distribuição: teste *t* de Student não pareado, teste de Mann Whitney e teste do qui-quadrado. Valores de p inferiores a 0,05 foram considerados estatisticamente significativos. A análise estatística foi realizada utilizando o software SSPS (versão 22.0).

## Resultados

Foram incluídos 134 pacientes no período de janeiro de 2014 a dezembro de 2016. Houve uma predominância de pacientes do sexo masculino, FEVE reduzida e diâmetros/volumes cavitários aumentados (Tabela 1). Pacientes não isquêmicos foram maioria, num total de 95 indivíduos (70,9%). Houve presença de realce tardio em 56 pacientes dos 95 com miocardiopatia não isquêmica (59%), com predomínio de realce mesocárdico e multifocal. Dentre os pacientes com miocardiopatia isquêmica, 34 pacientes (87%) apresentaram realce tardio, em sua maioria transmural.

**Tabela 1:** Características clínicas e funcionais da amostra

Características gerais	n (134)
Idade (anos) (DP)	50,2 (14,0)
Sexo masculino (%)	94 (70,1%)
Etiologia não-isquêmica	95 (70,9%)
Átrio esquerdo (cm) (DP)	3,9 (0,8)
Septo interventricular (cm) (DP)	0,8 (0,2)
Parede posterior (cm) (DP)	0,7 (0,2)
FEVD (%) (DP)	39,6 (15,9)
FEVE (%) (DP)	34,4 (17,9)
DDFVE (cm) (DP)	6,4 (1,2)
DSFVE (cm) (DP)	5,1 (1,6)
VDFVE (ml) (DP)	215,1 (96,2)
VDFVE indexado (ml/m^2^) (DP)	116,7 (51,9)
VSFVE (ml) (DP)	150,9 (93,7)
VSFVE indexado (ml/m^2^) (DP)	82,5 (52,3)
MC (g) (IQ)	88,5 (73,7; 114,0)
MC indexada (g/m^2^) (IQ)	49,0 (40,0; 62,5)
Hipertensão arterial	53 (39,6%)
Diabetes	21 (15,7%)
Doença coronária	33 (24,6%)
Doença renal crônica	13 (9,7%)
Tabagismo	20 (14,9%
Chagas	13 (9,7%)
Dislipidemia	7 (5,2%)

FEVE: Fração de ejeção do ventrículo esquerdo; FEVD: Fração de ejeção do ventrículo direito; DDFVE: Diâmetro diastólico final do ventrículo esquerdo; DSFVE: Diâmetro sistólico final do ventrículo esquerdo; VDFVE: Volume diastólico final do ventrículo esquerdo; VSFVE: Volume sistólico final do ventrículo esquerdo; MC: Massa cardíaca; DP: Desvio padrão; IQ: Intervalo interquartílico.

Fonte: Marques, 2019. Não esta nas referências

Valores de T1 miocárdico nativo elevados, quando analisado em relação ao VE, foram encontrados em pacientes com maior dilatação (p=0,007), maiores volumes ventriculares (p<0,01) e disfunção ventricular (p <0,001) (Tabela 2). Numa avaliação adicional de forma dicotomizada, considerando-se essas mesmas variáveis funcionais, manteve-se associação do valor do T1 miocárdico nativo, conforme exposto na Tabela 3. Quando realizada a análise de subgrupo dos pacientes não isquêmicos, as mesmas associações encontradas permaneceram presentes (Tabelas 3 e 4). Houve adequada concordância intraobservador na detecção de valores T1 elevados (Kappa 0,82; p = 0,001).

**Tabela 2:** Avaliação do mapa T1 miocárdico nativo com parâmetros funcionais

	T1 normal (ms)	T1 elevado (ms)	p
FEVE (%) (DP)	50,27 (16,3)	31,26 (16,5)	<0,001*
DDFVE (cm) (DP)	5,74 (1,2)	6,55 (1,2)	0,007*
DSFVE (cm) (DP)	3,95 (1,42)	5,32 (1,5)	<0,001*
VDFVE (ml) (IQ)	167,9 (62,5)	224,3 (99,1)	0,001*
VDFVE indexado (ml/m^2^) (DP)	89,8 (31,1)	122,0 (53,7)	0,001*
VSFVE (ml) (DP)	91,5 (60,2)	162,6 (94,8)	0,001*
VSFVE indexado (ml/m^2^) (DP)	47,9 (30,9)	89,3 (53,1)	0,001*
Massa (g) (IQ)	81,0 (66,0; 99,2)	89,5 (77,0; 119,5)	0,05†
Massa indexada (g/m^2^) (IQ)	41,5 (36,5; 52,5)	50,0 (40,5; 62,7)	0,025†

FEVE: Fração de ejeção do ventrículo esquerdo; DDFVE: Diâmetro diastólico final do ventrículo esquerdo; DSFVE: Diâmetro sistólico final do ventrículo esquerdo; VDFVE: Volume diastólico final do ventrículo esquerdo; VSFVE: Volume sistólico final do ventrículo esquerdo; MC: massa cardíaca; DP: desvio padrão; IQ: intervalo interquartílico.

* Teste T de student.

† Teste de Mann-Whitney

Fonte: Marques, 2019. Não está nas referências

**Tabela 3:** Avaliação do valor T1 miocárdico nativo com parâmetros funcionais na amostra geral e não isquêmicos

		Geral			Não Isquêmicos	
		T1 (ms)	p	T1 (ms)		p
FEVE (%) (DP)	>35%	1010,1 (46,6)	<0,001	1008,9 (43,7)		<0,001
	<35%	1053,4 (48,1)		1052,1 (48,1)		
DDFVE (cm) (DP)	Normal	1004,9 (48,1)	0,001	1010,8 (39,9)		0,03
	Dilatado	1042,7 (50,4)		1038,3 (53,4)		
DSFVE (cm) (DP)	Normal	989,0 (43,7)	<0,001	994,2 (37,7)		0,001
	Dilatado	1043,8 (49,0)		1040,3 (51,1)		
VDFVE indexado (ml/m^2^) (DP)	Normal	1021,3 (49,3)	0,001	1015,5 (46,0)		0,001
	Aumentado	1050,4 (50,8)		1049,2 (52,4)		
VSFVE indexado (ml/m^2^) (DP)	Normal	1000,7 (48,3)	<0,001	999,8 (42,5)		<0,001
	Aumentado	1048,5 (47,3)		1046,2 (49,5)		

FEVE: Fração de ejeção do ventrículo esquerdo; DDFVE: Diâmetro diastólico final do ventrículo esquerdo; DSFVE: Diâmetro sistólico final do ventrículo esquerdo; VDFVE: Volume diastólico final do ventrículo esquerdo; VSFVE: Volume sistólico final do ventrículo esquerdo; DP: desvio padrão. Teste T de student.

Fonte: Marques, 2019. Não está nas referências

**Tabela 4:** Avaliação do mapa T1 miocárdico nativo com parâmetros funcionais nos pacientes não isquêmicos

	T1 normal (ms)	T1 elevado (ms)	p
FEVE (%) (DP)	48,9 (16,6)	32,3 (17,9)	0,001*
DDFVE (cm) (DP)	5,9 (1,2)	6,6 (1,4)	0,035*
DSFVE (cm) (DP)	4,0 (1,5)	5,4 (1,7)	0,002*
VDFVE (ml) (IQ)	173,7 (66,8)	236,5 (112,8)	0,003*
VDFVE indexado (ml/m^2^) (DP)	92,2 (30,6)	122,7 (60,9)	0,001*
VSFVE (ml) (DP)	97,7 (63,3)	170,5 (107,9)	<0,001*
VSFVE indexado (ml/m^2^) (DP)	50,1 (31,2)	93,0 (60,2)	<0,001*
Massa (g) (IQ)	84,5 (66,7; 99,2)	91,0 (77,0; 129,0)	0,06†
Massa indexada (g/m^2^) (IQ)	42,0 (37,7; 52,5)	56,2 (42,0; 95,0)	0,02†

FEVE: Fração de ejeção do ventrículo esquerdo; DDFVE: Diâmetro diastólico final do ventrículo esquerdo; DSFVE: Diâmetro sistólico final do ventrículo esquerdo; VDFVE: Volume diastólico final do ventrículo esquerdo; VSFVE: Volume sistólico final do ventrículo esquerdo; MC: massa cardíaca; DP: desvio padrão; IQ: intervalo interquartílico.

* Teste T de student.

† Teste de Mann-Whitney.

Fonte: Marques, 2019. Não está nas referências

Quando analisado o T1 miocárdico nativo em relação ao perfil da IC, classificada conforme a FEVE, observou-se um maior valor T1 nos pacientes com FEVE < 35% (p < 0,001) (Tabela 5). Houve diferença significativa entre os grupos, com T1 mais elevado, quando comparado ICFER com ICFEL (p=0,004); e com ICFEP (p<0,001); assim como quando comparado ICFEL com ICFEP (p=0,02). Dos pacientes com ICFEP, 55,2% já apresentavam T1 elevado. Quando analisado em relação aos diâmetros e volumes cavitários, observados maiores valores nos pacientes com ICFER e ICFEL quando comparados com os ICFEP (p<0,01).

**Tabela 5:** Avaliação do mapa T1 miocárdico nativo com classificação de IC

	N	T1 normal (ms)	T1 elevado (ms)	p
ICFER	84	5 (6%)	79 (94%)	< 0,001*
ICFEL	21	4 (19%)	17 (81%)	
ICFEP	29	13 (45%)	16 (55%)	

ICFER: Insuficiência cardíaca com fração de ejeção reduzida; ICFEL: Insuficiência cardíaca com fração de ejeção limítrofe; ICFEP: Insuficiência cardíaca com fração de ejeção preservada.

* Teste de qui-quadrado.

Fonte: Marques, 2019.Não está nas referências

Quanto à etiologia da IC, foi observado que, independente da etiologia, há alta porcentagem de pacientes com T1 nativo elevado (89,7% nos isquêmicos e 81,1% nos não isquêmicos), com um maior valor T1 nos isquêmicos em relação aos não isquêmicos (p=0,004). Analisando especificamente o grupo de não isquêmicos, 13 pacientes tinham diagnóstico de cardiomiopatia chagásica, todos apresentam T1 nativo elevado (1077,1 ± 61,1ms) associado a FEVE reduzida (27,6 ± 16,8%), elevados DDFVE (7,1 ± 1,5cm), DSFVE (6,1 ± 1,7cm), VDFVE indexado (146,7 ± 52,3 ml/m^2^) e VSFVE indexado (112,7 ± 54,1 ml/m^2^).

Dentre as comorbidades clínicas avaliadas, observa-se associação estatística de maiores valores de T1, acima da faixa de normalidade, nos pacientes tabagistas (p=0,032). (Tabela 6)

**Tabela 6:** Avaliação do mapa T1 miocárdico nativo com comorbidades clínicas

	T1 normal	T1 elevado	p
Hipertensão arterial (%)	9 (17,0%)	44 (83,0%)	0,88
Diabetes (%)	1 (4,8%)	20 (95,2%)	0,11
Doença coronária (%)	4 (12,1%)	29 (87,9%)	0,44
Doença renal crônica (%)	0 (0%)	13 (100%)	0,09
Tabagismo (%)	0 (0%)	20 (100%)	0,03
Chagas (%)	1 (7,1%)	13 (92,9%)	0,09
Dislipidemia (%)	1 (14,3%)	6 (85,7%)	0,87

Teste de qui-quadrado.

Fonte: Marques, 2019. Não está nas referências

## Discussão

O presente estudo demonstra que, além de o Mapeamento T1 ser factível na prática clínica, existe associação do T1 miocárdico nativo com disfunção miocárdica, expressa por uma menor FEVE e maiores volumes e diâmetros intracavitários, independentemente da etiologia da cardiomiopatia.

A RMC permite a detecção de fibrose miocárdica difusa através do mapeamento T1 nativo e com alta concordância com a biópsia miocárdica.^6^ Um estudo recente publicado com 637 pacientes com CMD não isquêmica demonstrou que a presença de fibrose por mapeamento T1 nativo está relacionada ao desfecho combinado de mortalidade por todas as causas de IC (p <0,001), e na análise multivariada, é considerado como um preditor independente para esses desfechos (IC 1,06-1,15, p <0,001).^16^ Estudo prévio validou o uso do mapeamento T1 para confirmação de fibrose com excelente correlação (R = 0,95, p <0,001) entre o exame de RMC e a histologia, mas quando analisado comparando com o RTG, este último foi menos acurado na avaliação da fibrose intersticial difusa.^6^ Assim, o mapeamento T1 nativo do miocárdio é um método de imagem que permite detectar a presença de fibrose com maior precocidade que o RTG, que pode ser relacionada a um pior prognóstico.^22^

Entre as etiologias, no Brasil, há uma característica distinta em relação à prevalência e importância da doença de Chagas.^23^^,^^24^ No presente estudo, houve uma prevalência de 9,7% de cardiomiopatia Chagásica, que representa 13,7% dos pacientes não isquêmicos. Todos pacientes chagásicos tiveram valores nativos de T1 elevados, sendo observado um maior T1 nativo associado com menor FEVE, maior DDVE e VDFVE quando comparado com os demais não isquêmicos com T1 elevado, mas sem significância estatística. Estudos anteriores menores evidenciaram uma associação estatisticamente significativa (p<0,001) entre a presença de fibrose com piores desfechos nesses pacientes, principalmente relacionada a eventos arrítmicos.^23^^,^^24^ Em estudo anterior foi demonstrado que a probabilidade de taquicardia ventricular (TV) foi maior na presença de fibrose transmural por realce tardio, sendo um preditor de TV clínica (RR 4,1, p= 0,04).^23^

Há limitações que devem ser destacadas principalmente relacionadas ao modelo transversal do estudo. O tamanho da amostra foi limitado, o que impede a validação adequada das conclusões encontradas. Algumas patologias adicionais podem levar a alterações do T1, incluindo fibrose miocárdica difusa por outras causas, edema, inflamação e doenças infiltrativas. Como não foi realizado estudo de mapa T1 pós-contraste, o cálculo e avaliação do VEC não foi possível, o que não reduz a importância dos achados, uma vez que o T1 nativo demonstrou na literatura ser comparável ao VEC na quantificação de colágeno demonstrado histologicamente.^25^ Apesar de ter sido realizado e analisado de acordo com recomendações prévias, como o mapeamento T1 é um método relativamente novo, ainda requer padronização metodológica.^26^

## Conclusões

A utilização do mapa T1 miocárdico nativo é factível para avaliação da IC clínica, com correlação significativa a um pior perfil funcional. Houve associação direta entre maior valor nativo de T1 e piores parâmetros clínicos e funcionais, dentre eles uma menor fração de ejeção, diâmetros e volumes maiores do VE, independentemente da etiologia da cardiomiopatia. É importante ressaltar que, em pacientes com cardiopatia chagásica, patologia prevalente no Brasil, a mesma associação foi observada.
