# Supplementary material for: Estratificação da Insuficiência Cardíaca Clínica através do Mapa T1 Nativo: Experiência de um Serviço de Referência
Source: Arq Bras Cardiol. 2021 May 6;116(5):919–25. [Article in Portuguese] doi: 10.36660/abc.20190782 (PMC8121463; doi:10.36660/abc.20190782)
Supplement: Translation

# Translation: Clinical Heart Failure Stratification Through Native T1 Mapping: Experience of a Referral Service

**Authors:** Thiago dos Santos Silva Marques, André Maurício de Souza Fernandes, Roberto Nery Dantas, Robert W. Biederman, Ana Paula Marques de Oliveira Melo, Roque Aras

**Affiliations:** 1 São PauloSP Brazil Instituto do Coração do Hospital das Clínicas da Faculdade de Medicina da Universidade de São Paulo, São Paulo, SP - Brazil; 2 SalvadorBA Brazil Universidade Federal da Bahia - Faculdade de Medicina de Bahia, Salvador, BA - Brazil; 3 PittsburghPennsylvania USA Allegheny General Hospital, Pittsburgh, Pennsylvania - USA; 9 São PauloSP Brazil Hospital Heliópolis, São Paulo, SP - Brazil; 5 SalvadorBA Brazil Hospital Universitário Professor Edgard Santos, Salvador, BA - Brazil

**Keywords:** Heart Failure, Cardiomyopathy, Dilated, Ventricular Dysfunction, Left, Fibrosis, Diagnosis Imaging, Chagas Cardiomyopathy, Magnetic Resonance Spectroscopy/methods

## Abstract

**Background:**

Diffuse cardiac fibrosis is an important factor in the prognostic assessment of patients with ventricular dysfunction. Cardiovascular magnetic resonance imaging (CMR) native T1 mapping is highly sensitive and considered an independent predictor of all-cause mortality and heart failure (HF) development in patients with cardiomyopathy.

**Objectives:**

To evaluate the feasibility of native T1 mapping assessment in patients with HF in a cardiology referral hospital and its association with structural parameters and functional profile.

**Methods:**

Cross-sectional study with adult patients with HF NYHA functional classes I and II, ischemic and non-ischemic, followed in a referral hospital, who underwent CMR. Native T1 values were analyzed for structural parameters, comorbidities, etiology, and categorization of HF by left ventricular ejection fraction (LVEF). Analyses were performed with a significance level of 5%.

**Results:**

Enrollment of 134 patients. Elevated native T1 values were found in patients with greater dilation (1004.9 vs 1042.7ms, p = 0.001), ventricular volumes (1021.3 vs 1050.3ms, p <0.01) and ventricular dysfunction (1010.1 vs 1053.4ms, p <0.001), also present when the non-ischemic group was analyzed separately. Patients classified as HF with reduced ejection fraction had higher T1 values than those with HF and preserved ejection fraction (HFPEF) (992.7 vs 1054.1ms, p <0.001). Of those with HFPEF, 55.2% had higher T1.

**Conclusions:**

CMR T1 mapping is feasible for clinical HF evaluation. There was a direct association between higher native T1 values and lower ejection fraction, and with larger LV diameters and volumes, regardless of the etiology of HF.

## Introduction

Cardiac fibrosis has become an important factor in the prognostic evaluation of patients with ventricular dysfunction, considered as one of the consequences of left ventricular (LV) pathological remodeling,^1^ which plays an important role in myocardial response to injury. Fibrotic tissue leads to progression of heart failure (HF) and worse prognosis.^2^ Noninvasive imaging methods for quantitative assessment at an early stage of the presence and extent of myocardial fibrosis are necessary to better stratify the risk of HF and to monitor the effects of treatment.^3^

Cardiovascular magnetic resonance imaging (CMR), considered an effective tool for evaluating myocardial morphology and function, as well as tissue changes,^4^^–^^7^ has emerged as a first-line, noninvasive modality for investigation of etiology and prognosis in patients with myocardial dysfunction.^8^^,^^9^ Native T1 mapping is a fast, non-contrast method that aims to detect diffuse myocardial changes in a variety of cardiac conditions. It has a wide sensitivity for pathological changes, including detection of myocardial edema, infarction, ischemia, cardiomyopathies and diffuse fibrosis.^10^^–^^14^ Therefore, native T1 mapping provides an alternative imaging method for assessing the cardiac area at risk.^15^

A multicenter observational study showed that native T1 was a better predictor of worse outcomes in dilated cardiomyopathy (DCM) than the classic clinical parameters, showing that native T1 was the strongest independent predictor of all-cause mortality and development of HF.^16^^,^^17^

The severity of diffuse disease, assessed by the T1 map, maybe a pathophysiologically relevant parameter, since it is directly related to the progression of the disease and to the functional capacity of the remaining myocardium. The continuous nature of T1 values corresponds accurately to the rate of clinical events: the higher the native T1, the greater the risk of adverse events. These findings allow us to refine the current approach to risk stratification in patients with cardiomyopathies, especially DCM.^17^

Our study aims to evaluate the feasibility of native T1 mapping assessment in patients with HF in a cardiology referral hospital and its association with structural parameters and the functional profile of these patients.

## Methods

### Study Population

Patients were included in the period between 2012 and 2016. They were followed up at the HF outpatient clinic at Hospital Ana Nery, Salvador, Bahia, who were consecutively referred for CMR as part of the clinical care and diagnosis.

Patients aged ≥ 18 years with a diagnosis of HF, according to Framingham and/or Boston criteria, according to the Brazilian Guideline for Chronic and Acute Heart Failure, with functional classes I and II by the New York Heart Association (NYHA), with at least type II diastolic HF defined by transthoracic echocardiogram were consecutively selected. Multiple HF etiologies were divided into ischemic or non-ischemic groups, based on the documentation of myocardial infarction (MI), ischemia by some diagnostic method or presence of ischemic (transmural or subendocardial, following a coronary territory) late gadolinium enhancement (LGE) in CMR. In relation to Chagas cardiomyopathy, the diagnosis was considered in the presence of positive serology and after exclusion of ischemia.

All patients underwent routine examinations at the HF outpatient clinic, such as chest radiography, walking test and electrocardiogram, associated with the evaluation of a multidisciplinary team. All patients were followed up at the unit's Heart Failure service and used optimized drug therapy, associated or not with cardiac rehabilitation by the multidisciplinary team, according to the clinical criteria of the attending physician.

The work was approved by the institution's Ethics and Research Committee, as a subproject of the main work entitled “Characteristics of patients submitted to cardiovascular magnetic resonance at a referral hospital”.

### CMR Exam Acquisition Protocol and Image Evaluation

All CMR examinations were performed on a 1.5T Avanto full body scanner (Siemens Medical Solutions, Germany) using an 8-channel heart coil. Acquired images were performed to obtain 2D cine balanced SSFP stacks in two, three and four chambers, in addition to the short axis. The cine images were acquired during expiratory apnea (20 frames per cardiac cycle with cuts of 8mm thickness, FOV 300, matrix 208 Åx 80, BW 925 KHz / pixel). For analysis of the left ventricular function, the short axis was composed of a minimum of 8 and a maximum of 12 cuts, 8 mm thick and 2 mm wide.

Native T1 mapping images were performed without contrast injection in the mid-section of the LV through the Modified Look-Locker Inversion recovery (MOLLI) sequence, with electrocardiographic gating, 250 to 360 mm FOV; 192 × 122 to 192 × 183 matrix size. Slice thickness of 6-8 mm; 2.2 / 1.1ms ≈ TR / TE, flip angle 35°; Factor GRAPPA = 2; 17 heartbeats (collecting 3 + 3 + 5 samples). Due to the protocol used in the study, the calculation of extracellular volume (ECV) and post-contrast T1 mapping were not performed, since the use of contrast was optional and indicated only when necessary according to clinical evaluation.

The normal native myocardial T1 value for our sample was previously obtained through a pilot study with patients without comorbidities and structurally normal hearts, of the same institution / scanner, as recommended by Society for Cardiovascular Magnetic Resonance (SCMR).^18^ According to this evaluation, the average normal value considered for native myocardial T1 was 983.46 ± 34.38 ms.

All the exams were analyzed through the software cvi42 (Circle Cardiovascular Imaging Inc., Calgary, Canada) by a cardiovascular imaging specialist with more than 5 years of experience. After all the contours were drawn in the endocardial and epicardial borders of the LV short axis, in end systole and diastole, all functional variables were quantified, such as left ventricular ejection fraction (LVEF), left ventricular end-diastolic diameter (LVEDD), left ventricular end-diastolic (LVEDV) and end-systolic (LVESV) volumes and myocardial mass, all indexed to the body surface, according to recommended CMR reference values.^19^ To calculate the native T1 map, the edges of the tracings were made narrowly in order to avoid maximum contamination with the ventricular cavity or with epicardial fat, and in order to avoid areas with visibly identifiable late myocardial enhancement (Figure 1). The exams were analyzed by a single experienced professional.

**Figure 1:** Native T1 mapping calculation.

Source: Marques, 2019.

Native T1 values obtained were analyzed in relation to clinical comorbidities, structural parameters, etiology and HF categorization. HF was categorized into: 1) HFrEF (heart failure with reduced EF), EF<40%; 2) HFmrEF (heart failure with mid-range EF), EF 40-49% and; 3) HFpEF (heart failure with preserved EF), EF ≥ 50%.^20^^,^^21^

### Statistical Analysis

The collected data was described through averages and standard deviation for normal distribution variables; and median and interquartile range for the others. Categorical variables were described in absolute numbers and percentages. Variable normality was tested using the Kolmogorov-Smirnov. Statistical tests were performed according to the type of variable and distribution normality: unpaired Student's t-test, Mann Whitney test and chi-square test. P values less than 0.05 were considered statistically significant. Statistical analysis was performed using SSPS software (version 22.0).

## Results

We included 134 patients from January 2014 to December 2016. There was a predominance of male patients, reduced LVEF, and increased cavity diameters/volumes (Table 1). Non-ischemic patients were the majority, in a total of 95 individuals (70.9%). There was late enhancement in 56 patients out of 95 with non-ischemic cardiomyopathy (59%), with a predominance of mesocardial and multifocal enhancement. Among patients with ischemic cardiomyopathy, 34 patients (87%) had delayed enhancement, most of them transmural.

**Table 1:** Population's clinical and functional characteristics

General features	n (134)
Age (years) (SD)	50.2 (14.0)
Male gender (%)	94 (70.1%)
Non-ischemic etiology	95 (70,9%)
Left atrium (cm) (SD)	3,9 (0,8)
Interventricular septum (cm) (SD)	0,8 (0,2)
Posterior wall (cm) (SD)	0,7 (0,2)
RVEF (%) (SD)	39,6 (15,9)
LVEF (%) (SD)	34.4 (17.9)
LVEDD (cm) (SD)	6.4 (1.2)
LVESD (cm) (SD)	5.1 (1.6)
LVEDV (ml) (SD)	215.1 (96.2)
LVEDV index (ml/m^2^) (SD)	116.7 (51.9)
LVESV (ml) (SD)	150.9 (93.7)
LVESV index (ml/m^2^) (SD)	82.5 (52.3)
MM (g) (IR)	88.5 (73,7; 114,0)
MM index (g) (IR)	49.0 (40,0; 62,5)
Hypertension	53 (39.6%)
Diabetes	21 (15.7%)
Coronary artery disease	33 (24.6%)
Chronic renal failure	13 (9.7%)
Smoking	20 (14.9%)
Chagas Disease	13 (9.7%)
Dyslipidemia	7 (5.2%)

RVEF: right ventricular ejection fraction; LVEF: left ventricular ejection fraction; LVEDD: Left ventricular end-diastolic diameter; LVESD: left ventricular end-systolic diameter; MM: myocardial mass; LVEDV: left ventricular end-diastolic volume; LVESV: left ventricular end-systolic volume; SD: standard deviation; IR: interquartile range.

Source: Marques, 2019

Elevated native myocardial T1 values, when analyzed in relation to the left ventricle, were found in patients with greater dilation (p = 0.007), larger ventricular volumes (p <0.01) and ventricular dysfunction (p <0.001) (Table 2). In an additional dichotomized evaluation, considering these same functional variables, the associations of the native myocardial T1 value were maintained, as shown in Table 3. When the subgroup analysis of non-ischemic patients was performed, the same associations found remained present (Tables 3 and 4). There was adequate intraobserver agreement in detecting elevated T1 values (Kappa 0.82; p = 0.001).

**Table 2:** Evaluation of native T1 values with functional parameters

	Normal T1 (ms)	Abnormal T1 (ms)	p
LVEF (%) (SD)	50.27 (16.3)	31.26 (16.5)	<0.001*
LVEDD (cm) (SD)	5.74 (1.2)	6.55 (1.2)	0.007*
LVESD (cm) (SD)	3.95 (1.42)	5.32 (1.5)	<0.001*
LVEDV (ml) (SD)	155.0 (83.5)	200.0 (107.5)	0.001*
LVEDV index (ml/m^2^) (SD)	85.5 (47.0)	109.0 (49.8)	0.001*
LVESV (ml) (SD)	79.0 (72.3)	147.5 (102.8)	0.001*
LVESV index (ml/m^2^) (SD)	40.5 (36.0)	82.5 (57.5)	0.001*
MM (g) (IR)	81.0 (66.0; 99.2)	89.5 (77,0; 119.5)	0.05†
MM index (g/m^2^) (IR)	41.5 (36.5; 52.5)	50.0 (40.5; 62.7)	0.025†

LVEF: left ventricular ejection fraction; LVEDD: Left ventricular end-diastolic diameter; LVESD: left ventricular end-systolic diameter; MM: myocardial mass; LVEDV: left ventricular end-diastolic volume; LVESV: left ventricular end-systolic volume; SD: standard deviation; IR: interquartile range.

* Student's T test.

† Mann-Whitney test.

Source: Marques, 2019.

**Table 3:** Evaluation of native myocardial T1 values with functional parameters in the general and non-ischemic population

		General			Non-Ischemic	
		T1 (ms)	p	T1 (ms)		p
LVEF (%) (SD)	>35%	1010.1 (46.6)	<0.001	1008.9 (43.7)		<0.001
	<35%	1053.4 (48.1)		1052.1 (48.1)		
LVEDD (cm) (SD)	Normal	1004.9 (48.1)	0.001	1010.8 (39.9)		0.03
	Dilated	1042.7 (50.4)		1038.3 (53.4)		
LVESD (cm) (SD)	Normal	989.0 (43.7)	<0.001	994.2 (37.7)		0.001
	Dilated	1043.8 (49.0)		1040.3 (51.1)		
LVEDV index (ml/m^2^) (SD)	Normal	1021.3 (49.3)	0.001	1015.5 (46.0)		0.001
	Increased	1050.4 (50.8)		1049.2 (52.4)		
LVESV index (ml/m^2^) (SD)	Normal	1000.7 (48.3)	<0.001	999.8 (42.5)		<0.001
	Increased	1048.5 (47.3)		1046.2 (49.5)		

LVEF: left ventricular ejection fraction; LVEDD: left ventricular end-diastolic diameter; LVESD: left ventricular end-systolic diameter; LVEDV: left ventricular end-diastolic volume; LVESV: left ventricular end-systolic volume; SD: standard deviation;

* Student's T test.

**Table 4:** Evaluation of native myocardial T1 values with functional parameters in non-ischemic patients

	Normal T1 (ms)	Abnormal T1 (ms)	p
LVEF (%) (SD)	48.9 (16.6)	32.3 (17.9)	0.001*
LVEDD (cm) (SD)	5.9 (1.2)	6.6 (1.4)	0.035*
LVESD (cm) (SD)	4.0 (1.5)	5.4 (1.7)	0.002*
LVEDV (ml) (SD)	173.7 (66.8)	236.5 (112.8)	0.003*
LVEDV index (ml/m^2^) (SD)	92.2 (30.6)	122.7 (60.9)	0.001*
LVESV (ml) (SD)	97.7 (63.3)	170.5 (107.9)	<0.001*
LVESV index (ml/m^2^) (SD)	50.1 (31.2)	93.0 (60.2)	<0.001*
MM (g) (IR)	84.5 (66.7; 99.2)	91.0 (77.0; 129.0)	0.06†
MM index (g/m^2^) (IR)	42.0 (37.7; 52.5)	56.2 (42.0; 95.0)	0.02†

LVEF: left ventricular ejection fraction; LVEDD: left ventricular end-diastolic diameter; LVESD: left ventricular end-systolic diameter; MM: myocardial mass; LVEDV: left ventricular end-diastolic volume; LVESV: left ventricular end-systolic volume; SD: standard deviation; IR: interquartile range.

* Student's T test.

† Mann-Whitney test.

Source: Marques, 2019

When analyzing native myocardial T1 in relation to the HF profile, classified according to LVEF, a higher T1 value was observed in patients with LVEF <35% (p <0.001) (Table 5). There was a significant difference between the groups, with higher T1, when comparing HFrEF with HFmrEF (p = 0.004); and with HFpEF (p <0.001); as compared to HFmrEF with HFpEF (p = 0.02). Of the patients with HFpEF, 55.2% already had elevated T1. When analyzed in relation to diameters and cavity volumes, higher values were observed in patients with HFrEF and HFmrEF when compared with HFpEF (p <0.01).

**Table 5:** Association of native T1 values with heart failure classification

	N	Normal T1 (ms)	High T1 (ms)	p
HFrEF	84	5 (6%)	79 (94%)	< 0.001
HFmrEF	21	4 (19%)	17 (81%)	
HFpEF	29	13 (45%)	16 (55%)	

HFrEF: heart failure with reduced ejection fraction; HFmrEF: heart failure middle range ejection fraction; HFpEF: heart failure with preserved ejection fraction.

chi-square test

Source: Marques, 2019

Considering HF etiology, regardless of etiology, there was a high percentage of patients with elevated native T1 (89.7% in ischemic and 81.1% in non-ischemic), with a higher T1 value in ischemic patients compared to non-ischemic (p = 0.004). Specifically analyzing the non-ischemic group, 13 patients were diagnosed with Chagas cardiomyopathy, all presenting elevated native T1 (1077.1 ± 61.1ms) associated with reduced LVEF (27.6 ± 16.8%), high LVEDD (7.1 ± 1.5cm), LVESD (6.1 ± 1.7cm), indexed LVEDV (146.7 ± 52.3 ml/m^2^) and indexed LVESV (112.7 ± 54.1 ml/m^2^).

Among the comorbidities evaluated, there was a statistical association of higher T1 values, above the normal range, in smokers (p = 0.032). (Table 6)

**Table 6:** Native T1 values association with clinical comorbidities

	Normal T1	Abnormal T1	p
Hypertension (%)	9 (17.0%)	44 (83.0%)	0.88
Diabetes (%)	1 (4.8%)	20 (95.2%)	0.11
Coronary artery disease (%)	4 (12.1%)	29 (87.9%)	0.44
Chronic renal failure (%)	0 (0%)	13 (100%)	0.09
Smoking (%)	0 (0%)	20 (100%)	0.03
Chagas Disease (%)	1 (7.1%)	13 (92.9%)	0.09
Dyslipidemia (%)	1 (14.3%)	6 (85.7%)	0.87

chi-square test.

Source: Marques, 2019

## Discussion

The present study demonstrates CMR native T1 mapping feasibility in clinical practice with an association with myocardial dysfunction, expressed by lower LVEF and larger ventricular volumes and diameters, regardless of the etiology of the cardiomyopathy.

CMR allows the detection of diffuse myocardial fibrosis through T1 mapping, with high agreement with myocardial biopsy.^6^ A recently published study of 637 non-ischemic DCM patients demonstrated that the presence of fibrosis by native T1 mapping is related to the combined outcome of all-cause mortality and HF (p <0.001), and in the multivariate analysis, it is considered an independent predictor for these outcomes (CI 1.06-1.15, p <0.001).^16^ A previous study validated the use of T1 mapping to confirm fibrosis, with an excellent correlation (R = 0.95, p <0.001) between CMR examination and histology, and when analyzed in comparison with LGE, the latter was less accurate in the evaluation of diffuse interstitial fibrosis.^6^ Thus, native T1 mapping is an imaging method that allows the detection of fibrosis with greater precocity than the LGE, which is related to a worse prognosis.^22^

Among the etiologies in Brazil, there is a distinct characteristic regarding the prevalence and importance of Chagas disease.^23^^,^^24^ In the present study, there was a prevalence of 9.7% of Chagas cardiomyopathy, which represents 13.7% of non-ischemic patients. All these patients had elevated native T1 values, with a higher observed native T1 associated with lower LVEF, higher LVEDD and LVEDV when compared to the other non-ischemic T1-elevated patients, but without statistical significance. Fewer previous studies have shown a statistically significant association (p <0.001) between the presence of fibrosis with worse outcomes in these patients, mainly related to arrhythmic events.^23^^,^^24^ In a previous study, the risk of ventricular tachycardia (VT) was higher in the presence of transmural fibrosis by LGE, being a predictor of clinical VT (RR 4.1, p = 0.04).^23^

There are some limitations worth noting, mainly related to the cross-sectional model of the study. The sample size was limited, which precludes proper validation of the results. Some additional pathologies may lead to T1 changes, including diffuse myocardial fibrosis from other causes, edema, inflammation, and infiltrative diseases. As no post-contrast T1 mapping study was performed, the calculation and evaluation of the ECV was not possible, which does not reduce the importance of the findings, since native T1 has been shown in the literature to be comparable to ECV in quantification of histologically demonstrated collagen.^25^ Although it was performed and analyzed according to previous recommendations, as T1 mapping is a relatively new method, it still requires methodological standardization.^26^

## Conclusions

Native myocardial T1 mapping is feasible for clinical HF assessment, with significant correlation to worse functional profiles. There was a direct association between a higher native T1 value and worse clinical and functional parameters, including a lower ejection fraction, larger LV diameters and volumes, regardless of the etiology of cardiomyopathy. Importantly, in patients with Chagas heart disease, a pathology prevalent in Brazil, the same association was observed.
